# An electronic warning system helps to reduce the time to diagnosis of sepsis

**DOI:** 10.1186/cc14705

**Published:** 2015-09-28

**Authors:** Miriam Cristine V Machado, Álvaro Koenig, Geonice Sperotto, Glauco A Westphal

**Affiliations:** 1 Centro Hospitalar Unimed, Joinville, SP, Brazil

## Introduction

Reducing the time for the diagnosis of sepsis is a critical component to reduce the mortality rate related to severe sepsis and septic shock. The use of electronic warning devices may help in speeding the identification of sepsis risk patients.

## Objective

To evaluate the effect of an electronic alert system for the identification of septic patients on the time to antibiotic administration and mortality.

## Methods

An observational study that analyzed 480 patients with severe sepsis and septic shock in the period 2010-2014. An automatic warning system was implemented in 2010 to allow the identification of patients at risk for sepsis. This system sent automatic e-alerts for nurses of the wards through mobile devices. The Wilcoxon test and chi-square test were used for data analysis. *p *<0.05 was considered significant.

## Results

After implementation of the automated system, the time between screening and diagnosis decreased over the 4-year period (2010: 3:30 hours, 2011: 1:50 hours, 2012: 1:26 hours, 2013: 1:18 hours, 2014: 1:31 hours, *p *= 0.02). The total time between screening and antibiotic administration also reduced (2010: 5:36 hours, 2011: 3:00 hours, 2012: 2:30 hours, 2013: 3:03 hours, and 2014: 3:28 hours, *p *<0.02), with a concomitant mortality rate reduction (2010: 38.1 %, 2011: 29.3 %, 2012: 25.3 %, 2013: 19.6 % and 2014: 24.1 %, *p *= 0.03). Comparing survivors and nonsurvivors, we observed that the speed in screening patients at risk was similar (survivors: 2:22 ± 4:32 vs. nonsurvivors: 2:29 ± 5:06 p.m., *p *= 0.82). The variable "antibiotics in less than 1 hour" did not differ between survivors and nonsurvivors (161/350; 46 % vs. 55/130; 42.3 %, *p *= 0.47). However, nonsurvivors were older (69.5 ± 15 years vs. 55.7 ± 21.2 years, *p *<0.001), more sick (APACHE II score: 27.0 ± 8.1 vs. 19 ± 9.2, *p *<0.001) and showed higher levels of lactate at diagnosis (3.3 ± 2.9 mmol/l vs. 2.4 ± 1.7 mmol/l, *p *<0.01). At the end of 6 hours the lactate level was similar (survivors: 3.1 ± 3.8 mmol/l vs. non-survivors: 0.4 ± 1.9 mmol/l, *p *= 0.09).

## Conclusion

The electronic warning system helped to reduce the time necessary to perform the diagnosis of sepsis and the time to antibiotics. There was an association among the decrease in the time to diagnosis and mortality reduction. With the reduction in sepsis diagnosis time, intrinsic variables of patients seem to gain more weight in the risk of death associated with severe sepsis and septic shock.

